# A Sweet Talk: The Molecular Systems of Perineuronal Nets in Controlling Neuronal Communication

**DOI:** 10.3389/fnint.2017.00033

**Published:** 2017-12-01

**Authors:** Heleen M. van 't Spijker, Jessica C. F. Kwok

**Affiliations:** ^1^Department of Clinical Neurosciences, John van Geest Centre for Brain Repair, University of Cambridge, Cambridge, United Kingdom; ^2^Faculty of Biological Sciences, School of Biomedical Sciences, University of Leeds, Leeds, United Kingdom; ^3^Czech Academy of Sciences, Institute of Experimental Medicine, Centre of Reconstructive Neurosciences, Prague, Czechia

**Keywords:** perineuronal nets, hyaluronan, chondroitin sulfates, plasticity, neuronal communication, interneurons

## Abstract

Perineuronal nets (PNNs) are mesh-like structures, composed of a hierarchical assembly of extracellular matrix molecules in the central nervous system (CNS), ensheathing neurons and regulating plasticity. The mechanism of interactions between PNNs and neurons remain uncharacterized. In this review, we pose the question: how do PNNs regulate communication to and from neurons? We provide an overview of the current knowledge on PNNs with a focus on the cellular interactions. PNNs ensheath a subset of the neuronal population with distinct molecular aspects in different areas of the CNS. PNNs control neuronal communication through molecular interactions involving specific components of the PNNs. This review proposes that the PNNs are an integral part of neurons, crucial for the regulation of plasticity in the CNS.

## Introduction

Perineuronal nets (PNNs) were first described by Camillo Golgi as a reticular structure that enveloped nerve cells (Spreafico et al., 1999). They are a hierarchical assembly of proteoglycans and a selection of proteins. PNNs start with a meshwork of hyaluronan backbone synthesized by hyaluronan synthase (HAS), which is expressed on the surface of neurons (Brückner et al., 1993). The hyaluronan backbone provides a scaffold for the attachment of proteoglycans, including chondroitin sulfate proteoglycans (CSPGs) from the lectican family aggrecan, brevican, versican, and neurocan (Day and Prestwich, 2002). Proteoglycans are a family of glycans with glycosaminoglycan chains attached to a core protein. The majority of the glycosaminoglycan chains are chondroitin sulfate (CS) chains, which exist in different sulfation patterns (Deepa et al., 2006). A di-sulfation pattern occurs in CS-D and CS-E, while a single sulfation pattern occurs in CS-A and CS-C (Miyata and Kitagawa, 2015). The attachment of the proteoglycans to the hyaluronan backbone is stabilized by link proteins, the hyaluronan and proteoglycan link protein (HAPLN) family (Day and Prestwich, 2002). Tenascin R (TN-R) links the CSPGs together at their C-terminals (Morawski et al., 2014). Although the basic components are similar, the PNNs are not identical in each brain region, the quantities and precise composition of the components varies (Dauth et al., 2016). Although many of the components of the PNNs have been identified, more may exist and the details of their interactions remain unknown.

The PNNs appear around a subset of neurons and regulate plasticity, the capacity of neurons to adjust their synapses based on inhibitory or excitatory signals, after the closure of critical periods (Pizzorusso et al., 2002; Carulli et al., 2005; Kwok et al., 2015). The critical period is a period of high plasticity during development when neuronal networks develop and eventually consolidate their connections (Hensch, 2004). Critical periods in distinct brain regions occur at different ages. When the critical period of a neuronal network ends, the PNNs form and reduce plasticity of the neurons they envelope (Kwok et al., 2015). Removal of the PNNs by an enzyme called chondroitinase ABC (ChABC), which digests the CS chains and the hyaluronan backbone, reactivates plasticity (Pizzorusso et al., 2002; Grøndahl et al., 2011). A more specific enzyme, *Streptomyces* hyaluronidase, is applied to digest specifically the hyaluronan in the PNNs and also reactivates plasticity (Happel et al., 2014).

In this review, we address the question of the neuronal identity of PNN neurons in each brain region. These neurons are mostly fast spiking interneurons, which are key to the regulation of plasticity. Furthermore, we provide molecular mechanisms by which the PNNs influence the neuron it envelopes. We then describe the pathways through which the PNNs control the communication between neurons. The active participation of the PNNs with the properties of the wrapped neurons suggests that the PNNs are not just an extracellular coat, but an integral part of the neuron, which is crucial in regulating neuronal plasticity.

## Neuronal population of the PNNs

The PNNs are found around specific subgroups of neurons. We first discuss the populations of neurons enwrapped by the PNNs in different regions of the central nervous system (CNS) (Table 1) and subsequently, the recurring characteristics of the neurons enveloped with PNNs.

**Table 1:** The identities of PNN neurons in the central nervous system.

**CNS region**	**Neuron type**	**Location**	**References**
Cortex	GABAergic inhibitory interneurons, inhibitory pyramidal cells positive for parvalbumin (Härtig et al., 1999), specifically the mbpC positive parvalbumin neurons (Rossier et al., 2015).	Motor cortex, sensory cortex, prefrontal cortex, temporal cortex, layer 2-5 (Brückner et al., 1999), in visual cortex mostly 4-5, in mEC mostly 2-3 (Lensjø et al., 2017a).	Brückner et al., 1999; Härtig et al., 1999; Rossier et al., 2015; Lensjø et al., 2017a
Amygdala	Parvalbumin and calbindin positive inhibitory interneurons (Härtig et al., 1995) and excitatory neurons positive for CaMKII (Morikawa et al., 2017).	Lateral and basolateral nuclei (Morikawa et al., 2017).	Härtig et al., 1995; Morikawa et al., 2017
Hippocampus	Basket cells and bistratified neurons with high parvalbumin levels (Yamada et al., 2015). Excitatory pyramidal cells (Carstens et al., 2016).	Highest in CA2 (Lensjø et al., 2017a) CA1 and CA3 (Kochlamazashvili et al., 2010), dentate gyrus (Jansen et al., 2017).	Kochlamazashvili et al., 2010; Yamada et al., 2015; Carstens et al., 2016; Jansen et al., 2017; Lensjø et al., 2017a
Cerebellum	Excitatory Golgi neurons (Carulli et al., 2006) and Purkinje cells (Mabuchi et al., 2001) positive for parvalbumin.	Cerebellar cortex (Mabuchi et al., 2001) and nuclei (Lafarga et al., 1984; Blosa et al., 2016).	Lafarga et al., 1984; Mabuchi et al., 2001; Carulli et al., 2006; Blosa et al., 2016
Spinal cord	Large interneurons, 30% of motoneurons (Smith et al., 2015).	30% of motoneurons in ventral horn, 20% of neurons in the dorsal horn (Galtrey et al., 2008).	Galtrey et al., 2008; Smith et al., 2015

### Cortex

In the cortex, PNN neurons occur in high density in the motor and sensory cortex, as well as in the prefrontal and the temporal cortex. They are mostly found in layers 2–5 of the cortex (Brückner et al., 1999). However, there is some variation between cortical regions in the layers in which the PNNs can be found: the visual cortex shows the PNNs mostly in layer 4 and the lower part of layer 5, while in the medial entorhinal cortex (mEC) the PNNs are mostly found in layer 2 and 3(Lensjø et al., 2017a). The time at which PNNs mature also varies between cortical regions, in the mEC, PNNs mature earlier than in the visual cortex. Maturation of PNNs in the mEC coincides with the maturation of the grid cell pattern at postnatal day 30 (Lensjø et al., 2017a), while maturation of the PNNs in the visual cortex occurs at postnatal day 42 (Ye and Miao, 2013). The majority of neurons surrounded by PNNs in the cortex are GABAergic interneurons, while a smaller number of neurons surrounded by PNNs are pyramidal cells (Härtig et al., 1999; Beebe et al., 2016). There is a high co-localization of the GABAergic neurons ensheathed by PNNs and parvalbumin (Baig et al., 2005), specifically the myosin binding protein C (mbpC) positive parvalbumin neurons, while the somatostatin parvalbumin positive neurons do not show PNNs (Rossier et al., 2015). Parvalbumin positive neurons are inhibitory interneurons and they form the largest population of PNN positive cells in the brain (Baig et al., 2005). They are fast spiking interneurons which regulate pyramidal neurons, which in turn project out of the cerebral cortex and provide excitatory signals. When PNNs are removed from the visual cortex with ChABC, inhibitory activity is indeed reduced (Lensjø et al., 2017b). Electrophysiological recordings display an altered excitatory-inhibitory balance which resemble a reset to a juvenile state of the cortex, with increased plasticity (Lensjø et al., 2017b).

In the visual cortex, the formation of the PNNs can be delayed by dark rearing (Pizzorusso et al., 2002). Deprivation of stimuli by dark rearing disturbs the formation of the PNNs, delays the closure of critical period (a period of high plasticity during development) and thus the maturation of neural circuits in the visual cortex (Pizzorusso et al., 2002, 2006; Hensch, 2004). Furthermore, plasticity in the visual cortex can be reopened in adults by removing PNNs using ChABC treatment (Pizzorusso et al., 2002). Similar results were found in the auditory cortex when treatment with hyaluronidase reopened the window of plasticity (Happel et al., 2014). This suggests CS and hyaluronan in the PNNs are important for controlling plasticity in the cortex. Moreover, in C6ST-1 knockin mice, in which the ratio of 4S:6S is reduced, both the formation of PNNs and the maturation of parvalbumin neurons are impaired (Miyata et al., 2012). This genetic mutation also delays the closure of the critical period. In HAPLN1 KO mice, which show a reduction in PNNs, long term depression (LTD) is facilitated (Romberg et al., 2013). LTD is a reduction of the efficacy of the neuronal synapse lasting long after the stimulus, a form of plasticity. These changes in plasticity enhance the long term memory of the HAPLN1 KO mice (Romberg et al., 2013). Interestingly, PNNs in the cortex are also necessary for fear learning, which also requires plasticity. However, instead of blocking plasticity, in fear learning PNNs need to be dynamically regulated to make plasticity possible. A recent study shows that 4 h after fear conditioning, mRNA of PNN components were upregulated and more cells were surrounded with PNNs (Banerjee et al., 2017). This finding indicates that cells may respond to episodes of plasticity by increasing their PNNs. Together, these results show that PNNs in the cortex surround parvalbumin positive GABAergic interneurons and pyramidal cells and it regulates the closure of critical periods of plasticity.

### Amygdala

Perineuronal nets (PNNs) in the amygdala ensheath parvalbumin and calbindin positive neurons (Härtig et al., 1995). Both parvalbumin and calbindin positive neurons are GABAergic interneurons and both innervate pyramidal neurons. PNNs are also found surrounding excitatory neurons in the amygdala; PNNs in this region co-localize with Ca^2+^/calmodulin-dependent protein kinase II (CaMKII) (Morikawa et al., 2017). These excitatory neurons are necessary for fear memory. When cFos was used to mark activity in neurons, a correlation was found between the level of activity in the PNN excitatory neurons and the amount of freezing seen in the behavior of the animal (Hisaoka et al., 2010). Moreover, the appearance of the PNNs in the amygdala coincides with a developmental switch in fear memory resilience (Gogolla et al., 2009). This developmental switch specifies a period when the brain changes from a young brain, able to erase fear memories, to a mature brain in which fear memories can no longer be erased. The removal of the PNNs with ChABC completely blocks the expression of fear memory, suggesting that the memory is erased, which is not normally possible in the adult brain when the neuronal circuit is mature and PNNs have formed (Gogolla et al., 2009). Interestingly, the PNNs in the amygdala are both necessary for the protection and renewal of fear memories (Gogolla et al., 2009). This indicates the PNNs are not simply blocking the plasticity of neurons, but are also a crucial component of the plasticity. Treatment with ChABC abolishes spontaneous recovery and renewal of conditioned fear memory, while it does not interfere with memory consolidation (Gogolla et al., 2009). In the amygdala, the PNNs envelope both inhibitory and excitatory interneurons and the development of PNNs in the amygdala coincides with a switch in fear memory regulation.

### Hippocampus

In the hippocampus, a high density of PNNs is found in the CA2 (Lensjø et al., 2017a), fewer PNNs are found in the CA1, CA3 (Kochlamazashvili et al., 2010) and the dentate gyrus (Jansen et al., 2017) The PNNs are found around basket cells and bistratified neurons with high parvalbumin levels (Yamada et al., 2015). These inhibitory interneurons control pyramidal cells in the local circuit. In the CA2, the PNNs are also found around the excitatory pyramidal neurons (Carstens et al., 2016). The PNNs on the excitatory neurons suppress plasticity of excitatory synapses. Removal of the PNNs with ChABC leads to synaptic potentiation and the excitatory postsynaptic current (EPSC) amplitude increases (Carstens et al., 2016).

In a mouse model where PNN components brevican, neurocan, TN-R, and Tenascin C were deleted, synaptic depression was found amplified in the dentate gyrus (Jansen et al., 2017). When neurons and astrocytes from this KO model are cultured, a reduction in frequency of both EPSCs and inhibitory postsynaptic currents (IPSCs) was observed (Geissler et al., 2013). These studies indicate that the deletion of the PNN components leads to a loss of synaptic plasticity in hippocampal neurons. Similarly, in a TN-R KO model, reduction of long term potentiation (LTP) was found in the CA1 region (Bukalo et al., 2001). The threshold for the induction for LTP is also increased and the basal excitatory synaptic transmission is elevated (Bukalo et al., 2007). LTP is a persistent strengthening of the synapse and a form of synaptic plasticity. The loss of the potential to strengthen synapses when TN-R, a component of the PNNs, is missing, shows the PNNs do not only block plasticity but regulate plasticity of neurons in various manners. Indeed, removal of the PNNs with hyaluronidase treatment reduces calcium currents, which are crucial for neuronal activity, in the CA3 and CA1 (Kochlamazashvili et al., 2010).

Similar to findings in the amygdala and the cortex, PNN removal with ChABC and hyaluronidase in the hippocampus disrupts fear memory formation (Hylin et al., 2013). The function of the PNNs as a regulator of plasticity is also demonstrated in status epilepticus, a prolonged state of seizure. After status epilepticus, a period of enhanced neuronal activity, PNNs are lost (McRae et al., 2012). This finding indicates the PNNs are regulated by neuronal activity. Status epilepticus causes synaptic reorganization in the hippocampus, which is possible due to PNN removal (McRae et al., 2012). These findings indicate the PNNs in the hippocampus surround both excitatory and inhibitory neurons and in both cases the PNNs are crucial for the control of plasticity.

### Cerebellum

In the cerebellum, the PNNs can be found in the cerebellar cortex (Mabuchi et al., 2001) as well as in lateral and basolateral nuclei (Lafarga et al., 1984; Blosa et al., 2016) of the cerebellum. The PNNs in the cerebellum envelope the large excitatory Golgi neurons (Carulli et al., 2006), which synapse on the granule cells. Thin and compact PNNs can also be found around the Purkinje cells (Mabuchi et al., 2001), which project to the deep cerebellar nuclei. Both Golgi neurons and Purkinje cells are positive for parvalbumin. When PNN formation is blocked by the removal of one of the link proteins, HAPLN1, the Purkinje cell terminal is enhanced (Foscarin et al., 2011). Furthermore, when animals are reared in an enriched environment, which leads to higher plasticity in the cerebellum, the formation of the PNNs is reduced (Foscarin et al., 2011). The formation of the PNNs is linked to the level of plasticity in the cerebellum.

### Spinal cord

In the spinal cord, the PNNs are mostly found around interneurons, as well as around 30% of the motoneurons (Smith et al., 2015) in the ventral horn and around 20% of the neurons in the dorsal horn (Galtrey et al., 2008). Thickness of PNNs in the spinal cord increases with exercise, which suggests higher synaptic activity can enlarge PNNs (Smith et al., 2015). Treatment with ChABC in the spinal cord reinstates plasticity, which allows for the recovery of forelimb functions after spinal cord injury (Galtrey et al., 2007; Wang et al., 2011). This suggests that PNNs also act as a brake on plasticity in the spinal cord. Interestingly, the removal of the PNNs only leads to recovery when it is accompanied by rehabilitation (Wang et al., 2011). New stimuli to the spinal cord are needed to make use of the reopening of the plasticity by the removal of the PNNs.

### Common features and divergence of PNN neurons across CNS regions

A review on the identities on PNN neurons in different regions of the CNS allows us to draw a comparison on the properties on PNN neurons. There are many parallels on the formation of PNNs from the various CNS regions. In the cerebellum, the CSPGs which form the PNNs are produced both by glial cells and by neurons (Carulli et al., 2006). However, only neurons have the mRNA for the hyaluronan synthases (HASs) which produce hyaluronan, the backbone of the PNNs. Similar to the findings in the cerebellum, in the spinal cord HAS, together with aggrecan, HAPLN1 and brain link protein 2 (BRAL2) are only expressed by neurons (Galtrey et al., 2008). In both regions many PNN components are produced by multiple cell types but only neurons produce HASs (Carulli et al., 2007; Galtrey et al., 2008). This similarity indicates there is a shared underlying mechanism for the formation of the PNNs although variation between regions can also be found. Indeed, recombinant expression of HASs in cell culture allows the formation of pericellular matrix similar to PNNs (Kwok et al., 2010; Giamanco and Matthews, 2012).

Divergence between PNNs in different regions is caused by variations in the components of the PNNs, such as aggrecan (Matthews et al., 2002). Aggrecan exists with different glycosylation patterns and subtypes of neurons produce aggrecan with different glycosylation patterns (Lander et al., 1997; Matthews et al., 2002). The components of the PNNs also have distinct spatial patterns in the brain (Dauth et al., 2016). For example, aggrecan, brevican, and Tn-R differ in their spatial patterns, brevican intensity is higher in the caudate-putamen than in the thalamus, whilst aggrecan, and Tenascin-R show lower intensity in the caudate-putamen than in the thalamus. It is likely the heterogeneity of the PNNs reflects the variety of functions carried out by neurons with PNNs.

Overall, across CNS regions, PNNs surround a variety of interneurons and regulates plasticity. Since PNNs are mostly found around inhibitory interneurons in the cortex, an initial proposal for PNN functions is that PNNs act as a regulator on inhibitory neurons to balance the inhibitory and excitatory inputs (Dityatev et al., 2010). However, this proposal has been adjusted due to the identification of PNNs on excitatory neurons. It has been proposed that PNNs surrounding excitatory synapses could lead to an early closure of critical period (Carstens et al., 2016). The fast spiking interneurons have a high capacity for plasticity and the consistent presence of PNNs on the fast spiking interneurons provides an extra “tool” for the regulation of plasticity.

## How do the PNNs influence neurons?

**Figure 1:**
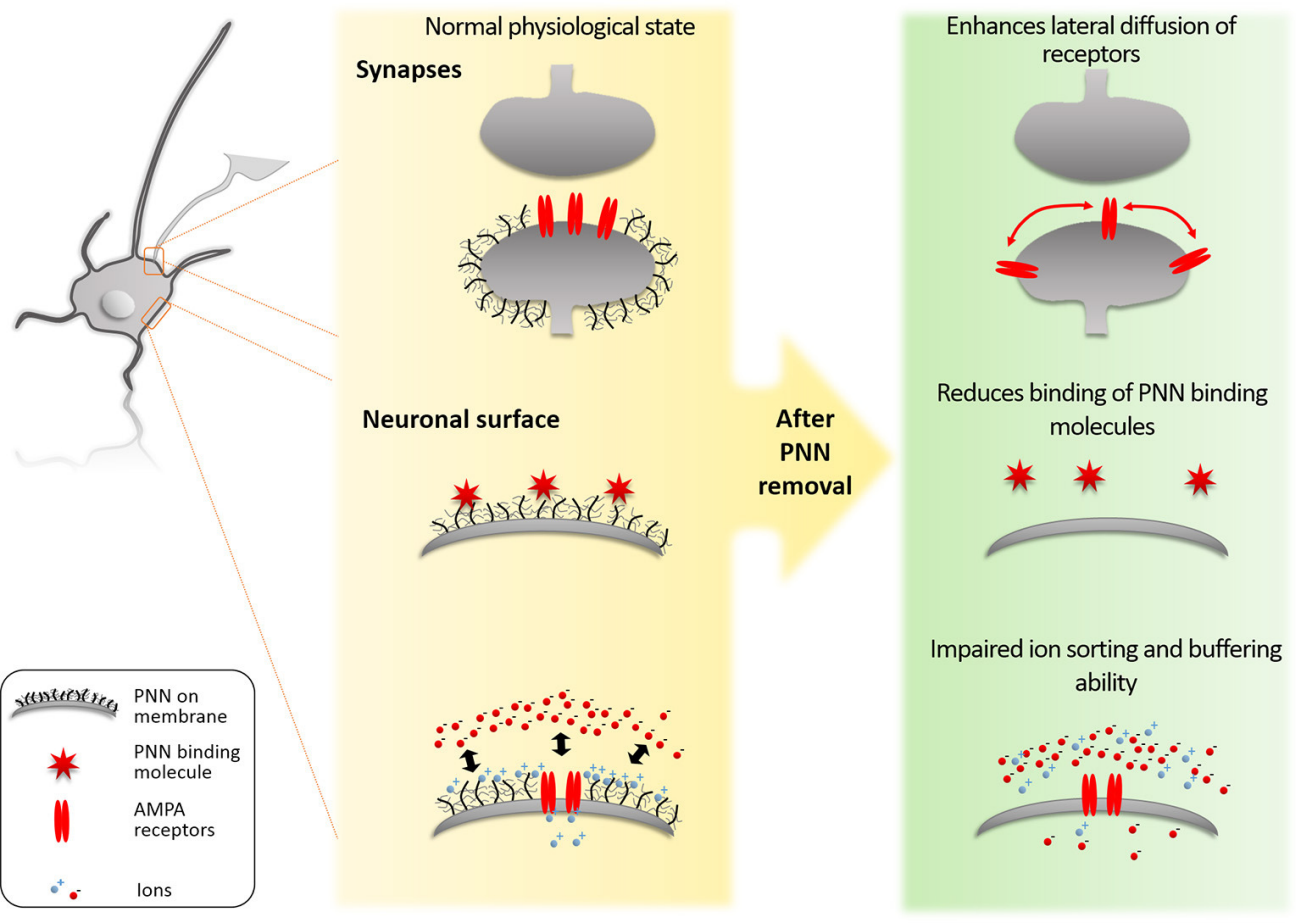
Molecular mechanisms of PNNs. (1) The PNNs block lateral diffusion of membrane bound proteins such as AMPA receptors. By this mechanism the PNNs limit synaptic plasticity. (2) The PNNs bind specifically to proteins such as Sema3A and Otx2. This binding regulates which ECM proteins can reach the PNN neuron and also present these proteins on its surface to signal to approaching axons from other neurons. (3) The PNNs act as a physical barrier for ion sorting and buffering on the neuronal surface. The high negative charge of the PNNs repels anionic ions/molecules (such as reactive oxygen) to reach the neuronal surface, while it attracts the cationic ions/molecules and creates a reservoir for fast buffering of ions required for the synaptic function and to prevent oxidative stress induced by Fe^3+^.

Perineuronal nets (PNNs) influence neurons through different mechanisms: it acts as (1) a physical barrier between the neuron and the soluble extracellular matrix; (2) a binding partner for molecules that interact with neurons; and (3) a barrier to prevent lateral mobility of molecules on the neuronal membrane (Figure 1). Here, we discuss these processes and their effects on the function of neurons and the region regulated by those neurons.

### Physical barrier

The PNNs act as a physical barrier between the neuron and the extracellular space. It acts as a local buffering reservoir for ions (Morawski et al., 2015), protects the neuron from oxidative stress (Suttkus et al., 2014) and toxic protein species (Miyata et al., 2007). Both hyaluronan and CSs are highly negatively charged molecules, and their presence allows PNNs to act as a local buffer for cations close to the synapse (Härtig et al., 1999). The PNNs mostly envelope fast spiking interneurons, which have high activity at their synapses and therefore need buffering for the cations involved in the neurotransmission. Using Fe^3+^ as a cationic probe, PNNs have been detected as a structure with high local charge density in patches on neuronal surfaces on tissue slices (Morawski et al., 2015). The concentration of negative charge in the PNNs is high enough for the PNNs to perform ion sorting properties on the neuronal surface (Morawski et al., 2015). The PNNs can also buffer ions, such as Fe^3+^, to prevent oxidative damage in the neuronal microenvironment (Suttkus et al., 2014). This was shown in the aggrecan, TN-R and HAPLN1 triple KO model, in which PNN formation is impaired (Suttkus et al., 2014). The study showed that PNNs protect neurons from oxidative stress induced by FeCl_3_. After a deletion of aggrecan, TN-R or HAPLN1, protection against the oxidative stress caused by FeCl_3_ is lost (Suttkus et al., 2014). Similarly, PNN removal with ChABC makes parvalbumin neurons more vulnerable to oxidative stress (Cabungcal et al., 2013). The PNNs also protect neurons from the toxicity of amyloid beta (Miyata et al., 2007). Amyloid beta has a neurotoxic effect on neurons without PNNs, but not on neurons surrounded with PNNs. When the PNNs are removed with ChABC, neuronal death caused by amyloid beta is increased. Similar observation is made in human brains where PNN neurons are indeed protected from amyloid beta plaques (Morawski et al., 2012). These examples show that PNNs act as a physical barrier to molecules that can damage their underlying neurons.

Apart from acting as a barrier between the extracellular matrix molecules and the neurons, the PNNs also inhibit the growth of neurites and thus deters synapse formation. CSPGs, one of the main components of the PNNs, are strong inhibitory molecules highly up-regulated in the glial scar after CNS injury, they collapse growth cones and inhibit neural regeneration (Cheah et al., 2016; Shinozaki et al., 2016). Breaking down CSPGs with ChABC treatment improves the regeneration of axons (Shinozaki et al., 2016). The role as a plasticity brake of PNNs is partially attributed to the presence of CSPGs in the structure.

### Specific binding of proteins

The different components of the PNNs have specific binding capacities for proteins. Several growth factors, such as midkine and fibroblast growth factor 2, have been shown to bind specifically to chondroitin sulfate E (CS-E) which is enriched in PNNs compared to the soluble extracellular matrix (ECM) (Deepa et al., 2002). Similarly, the chemorepulsive molecule Semaphorin3a (Sema3A) binds to the PNNs via CS-E (Dick et al., 2013). Removal of the PNNs with ChABC removes Sema3A from the neuronal surface (Vo et al., 2013). The finding suggests that the PNNs present Sema3A to approaching axons from other neurons to allow Sema3A to function as a repulsive signaling molecule (de Winter et al., 2016).

TN-R carries a HNK-1 epitope, which allows it to bind GABA receptors (Bukalo et al., 2007). GABA receptors are key regulators of synaptic activity, so this binding capacity allows the PNNs to regulate the synapse. In TN-R KO animals, a higher calcium influx is observed (Bukalo et al., 2007), which shows a relation to plasticity of the synapse. The hyaluronan backbone of the PNNs has the capacity to modulate postsynaptic L-type calcium channels (Kochlamazashvili et al., 2010). Treatment with hyaluronidase indeed reduced calcium currents in the hippocampus (Kochlamazashvili et al., 2010). Another regulator of synaptic proteins is brevican, which regulates potassium channels and AMPA receptors (Favuzzi et al., 2017). This function allows brevican to regulate the activity dependent gating of parvalbumin interneuron function (Favuzzi et al., 2017). Brevican deficient mice indeed have impaired LTP in the CA1 of the hippocampus (Brakebusch et al., 2002).

The transcription factor orthodenticle homeobox 2 (Otx2), needs to be captured by PNNs to be internalized by the neuron (Beurdeley et al., 2012). This internalization is crucial for the maturation of parvalbumin positive neurons in the cortex and regulate plasticity. Treatment with ChABC removes the PNNs and reduces the amount of Otx2 inside the neuron. A recent study also demonstrates that the PNNs regulate the level of PV expression and thus the plasticity capacity of a neuron (Donato et al., 2013). In an animal model with a point mutation on the Otx2 gene, maturation of parvalbumin interneurons is delayed (Lee et al., 2017). Similar to the previous literature using OTX2 blocking peptide, the closure of critical period plasticity is also delayed in this model, confirming the importance of the binding of Otx2 to the function of PNNs in critical period plasticity. This suggests that PNNs affect neurons through its capacity to selectively bind growth factors, transcription factors and signaling molecules, which ultimately affect the neuronal functions.

In addition to the binding partners discussed above, there are other proteins which may play a similar role as Sema3A or OTX2 to the PNNs. Recently, the tumor necrosis factor-stimulated gene-6 (TSG-6) was identified in the glial scar (Coulson-Thomas et al., 2016). The expression of TSG-6 is up-regulated after spinal cord injury (Coulson-Thomas et al., 2016). TSG-6 binds to hyaluronan and stabilizes the hyaluronan matrices (Baranova et al., 2011). Since the backbone of PNNs consists of hyaluronan it is possible TSG-6 binds to the PNNs and has a stabilizing role comparable to its role in the glial scar. Other than TSG-6, another extracellular protein neuronal pentraxin 2 (Nptx2) has also been proposed as a PNN binding partner. ChABC treatment, which removes PNNs, removes Nptx2 from the surface of neurons (Chang et al., 2010). Since Nptx2 is involved in the regulation of critical periods, just like the PNNs, and ChABC removes Nptx2, it is likely the protein is a binding partner of the PNNs via the CS component (Gu et al., 2013). Nptx2 controls plasticity through clustering of α-amino-3-hydroxy-5-methyl-4-isoxazolepropionic acid (AMPA) receptors (Pelkey et al., 2015). It has been previously shown that the PNNs limit AMPA receptor movement (see the next section) (Frischknecht et al., 2009). PNNs may regulate AMPA receptors through the regulation of NPTX2. Both proteins are potential candidates for specific binding partners of the PNNs.

### Limitation to lateral mobility on the neuronal membrane

Perineuronal nets (PNNs) limit the mobility of membrane bound proteins on the neuronal surface. When the PNNs are removed from the neuronal surface using hyaluronidase in neuronal cultures, lateral diffusion of AMPA receptor subunits increases (Frischknecht et al., 2009). Whole-cell patch-clamp recordings showed that the induced increase in diffusion allows a fast exchange of desensitized receptors under high stimulation, which then leads to an increase in paired-pulse ratio, a form of short term synaptic plasticity (Frischknecht et al., 2009). The limitation to the lateral mobility of membrane bound proteins caused by the PNNs allow the PNNs to inhibit synaptic plasticity.

## Functional changes in the neuron

The molecular assembly of the PNNs leads to functional changes in the neurons they envelope: when the physical barrier function is lost, FeCl_3_ causes oxidative stress in the neuron (Suttkus et al., 2014); when the binding function of the PNNs is lost, proteins such as Otx2 no longer reach the neuron and thus affect its maturation (Beurdeley et al., 2012); and when the lateral mobility of neuronal membrane proteins is increased by the removal of PNNs, short-term plasticity is increased (Frischknecht et al., 2009). These molecular mechanisms demonstrate that the molecular properties and interactions of the PNNs are crucial for the function and identity of neurons. This also means that manipulation of PNNs can change the function of neurons. When the PNN formation is prevented by knocking out several of the PNN components, an increased number of synapses is formed (Geissler et al., 2013). However, the newly formed synapses do not function as normal synapses, because they show a reduced frequency of excitatory and inhibitory postsynaptic currents (Geissler et al., 2013). This indicates that the PNNs are necessary for effective synaptic signaling and stabilization of synapses. Interestingly, temporarily removing the PNNs with ChABC leads to increased sprouting of axons in the cerebellum (Corvetti and Rossi, 2005) and in the spinal cord (Barritt et al., 2006). Axonal sprouting is a form of structural neuronal plasticity, the axon grows in a new direction to make new synaptic connections possible. The PNNs normally block this form of neuronal plasticity. The temporary removal of the PNNs not only allows for the formation of new synapses, but the synapses can also be functional: ChABC treatment can promote recovery of forelimb function (Wang et al., 2011). Since the PNNs are necessary to stabilize synapses, the recovery of PNNs after temporary removal can be helpful to stabilize the newly formed connections. Knowledge of the molecular interactions of the PNNs can increase our capacity to subtly interfere with the PNNs to allow for treatment without permanent damage.

## The role of PNNs in communication between cells

Communication between neurons is regulated by the PNNs. The PNNs regulate which molecules produced by other neurons or glia reach the neuron, present molecules to other neurons and regulate cell signaling at the synapse. All processes will be described here and their influence on the functioning of the neuron will be discussed.

### Regulation of molecules accessing the neuron

As discussed earlier in this review, many of the components of the PNNs have binding capacities for specific proteins. The PNNs can apply this function to attract proteins to the neuron. This process has been investigated with Otx2. Otx2 relies on the PNNs to enter the neuron and influence plasticity (Beurdeley et al., 2012). Otx2 is expressed in the choroid plexus and spreads to other brain regions through the cerebrospinal fluid (Spatazza et al., 2013). When Otx2 reaches the visual cortex, it binds the PNNs and enters the neuron to regulate maturation and thus affect plasticity (Sugiyama et al., 2008). The Otx2 internalization is mediated through its binding to CS-D and CS-E in the PNNs. Consequently, when the PNN is removed with ChABC, the amount of Otx2 in the neurons is reduced (Beurdeley et al., 2012). This indicates that the communication between choroid plexus and the visual cortex relies on the PNNs to bind Otx2. The PNNs enable long distance communication between neurons by its binding specificity.

### Presentation of molecules to other neurons

The PNNs also regulate the signals which a neuron presents to its surrounding ECM and potentially other cells. Semaphorin 3A (Sema3A) binds the PNNs and potentiates the inhibition of PNNs to the growth of axons (Dick et al., 2013) and potentially to the formation of synapses. Inhibition of neurite outgrowth of dorsal root ganglion cultures on a surface of PNN glycans, is stronger when the surface of PNN glycans is mixed with Sema3A (Dick et al., 2013). This finding indicates the binding of Sema3A to the PNNs allows the PNNs to inhibit the approach of axons from other neurons toward the neuron it surrounds. In contrast, the PNNs block integrin activation and signaling to other neurons (Orlando et al., 2012). Removal of the PNN with ChABC allows activation of integrins which leads to enhanced spine motility (Orlando et al., 2012). In conclusion, the PNNs control communication of the neurons they surround toward other neurons through the altered presentation of protein signals.

### Regulation of cell signaling at the synapses

Neurons communicate through signaling at the synapse and the PNNs regulate the synapse with several methods. As described earlier in this review, PNNs acts as a physical barrier to limit AMPA receptor mobility on the neuronal membrane (Frischknecht et al., 2009). The limitation of the AMPA receptor mobility blocks them from leaving the synaptic cleft, which keeps the amount of AMPA receptors at the synapse constant. The physical barrier function of the PNNs thus reduces the capacity for plasticity.

The PNNs also regulate the synapse through parvalbumin. Many of the neurons surrounded by PNNs are parvalbumin expressing interneurons (Härtig et al., 1995; Baig et al., 2005; Yamada et al., 2015). Parvalbumin is a calcium binding protein which regulates short term synaptic plasticity, which was shown with electrophysiology experiments on a parvalbumin knockout mouse model (Caillard et al., 2000). It is likely parvalbumin regulates the short term plasticity by binding the calcium ions. In the mouse hippocampus, digestion of PNNs with ChABC leads to a decrease of parvalbumin levels inside neurons (Yamada et al., 2015). Both the amount of parvalbumin mRNA as well as the amount of the protein itself are reduced by ChABC injection into the brain. These results show that PNNs help in maintaining the amount of parvalbumin in the neuron which allows it to regulate plasticity.

The PNNs also block the formation of new synapses between neurons (Geissler et al., 2013). When the formation of the PNNs is decreased in cultured neurons by knockout of brevican, neurocan, TN-R, and Tenascin-C, the neurons show an increase in the amount of inhibitory synapses they form. Similarly, when the PNNs are removed with ChABC, synaptogenesis is increased between transplant and host neurons (Suzuki et al., 2007). The formation of new synapses allows neurons to form a new route to communicate. In conclusion, the PNNs regulate cell signaling at the synapse.

## Conclusion

Perineuronal nets (PNNs) are an integral part of a neuron and regulate communication between neurons. The PNNs are found in most brain regions and in each region, the PNNs envelope a limited group of neurons. In general, the PNNs are mostly formed around subpopulations of inhibitory neurons but also around some excitatory neurons. The PNN interneurons are mostly fast spiking neurons. It is likely these neurons have evolved to produce PNNs because they are in need of a tool to handle this high level of synaptic activity. PNNs allow the neuron to react to the stress of a high amount of inhibitory and excitatory stimuli.

The PNNs regulate plasticity through a variety of molecular interactions. They function as a physical barrier to block the entrance of toxic substances such as amyloid β. The components of PNNs bear a highly negative charge, which allows the PNNs to buffer Fe^3+^ and thus protect the neuron from oxidative stress. The negative charge also regulates ion sorting, which is crucial for fast spiking neurons that have a high utilization of ions. The PNNs have binding capacities for specific molecules, such as Otx2 and Sema3A. They also specifically bind several synaptic receptors and ion channels to regulate the synapse. Furthermore, the PNNs limit lateral mobility of membrane bound proteins. The limitation of lateral mobility affects synaptic proteins such as AMPA receptors. The localization of synaptic proteins is crucial for the efficiency of the synapse, which can in turn lead to plasticity. The three molecular mechanisms described here are mechanisms by which PNNs regulate plasticity.

The molecular interactions in which the PNNs are involved allow the PNNs to regulate communication between neurons. The PNNs regulate which molecules produced by other neurons reach the neuron through its selective binding properties. The PNNs also present signaling molecules to other neurons. Lastly, the PNNs are directly involved in the cell signaling taking place at the synapse. The different communication methods are essential for highly active interneurons to adapt to their surroundings. Interneurons process high amounts of input and fire at a high rate, which leads to high metabolic activity and the risk of oxidative stress. The interneurons produce PNNs to manage this high amount of activity. It is possible that PNNs enable neurons to synchronize their activity. Removal of PNNs with ChABC increases high frequency oscillations in the anterior angulate cortex (Steullet et al., 2014). In TN-R KO mice the hippocampal gamma oscillation are enhanced (Gurevicius et al., 2004). These findings indicate PNNs regulate the activity of whole fields of neurons, which means the regulatory effects on the level of the single neuron allow the neurons to cooperate. Moreover, the formation of PNNs in many regions coincides with the end of a critical period, which means PNNs allow neurons to regulate their whole region. The PNNs are multi-application tools for the neuron to regulate communication.

Further investigations into function of the PNNs need to focus on the PNNs as a tool which neurons actively apply to regulate communication. The PNNs form at the outermost surface of the neuron and can serve as an easily accessible target for plasticity treatment to be applied. This would facilitate the modification of neuronal communication. Currently, the enzymatic removal of PNNs by ChABC and hyaluronidase is widely applied as tools for PNN regulation but these are very harsh and non-specific treatments since they remove PNNs and the loose ECM completely. More subtle manipulations of the PNNs by changing the ratio of the different proteoglycans would potentially allow for fine modifications of neuronal plasticity without harming the neuron. In diseases which present with a loss of PNNs, stimulation of PNN formation by providing of PNN components could help to protect neurons. Application of binding partners of PNNs might be another approach to modulate PNN functions without damaging it. Treatment designed to alter the PNNs would not have to enter cells, which makes the PNNs an accessible molecular structure for treatments. Since the PNNs are involved in a variety of diseases, such as schizophrenia (Pantazopoulos et al., 2010), epilepsy (Arranz et al., 2014), Alzheimer's disease (Brückner et al., 1999) and spinal cord injury (Bradbury et al., 2002), such an accessible tool for neuronal communication is very promising.

## Author contributions

Both HvS and JK contributed equally to the idea and the writing of the review.

### Conflict of interest statement

The authors declare that the research was conducted in the absence of any commercial or financial relationships that could be construed as a potential conflict of interest.
